# Limited Impact of Murine Placental MDR1 on Fetal Exposure of Certain Drugs Explained by Bypass Transfer Between Adjacent Syncytiotrophoblast Layers

**DOI:** 10.1007/s11095-022-03165-6

**Published:** 2022-01-26

**Authors:** Arimi Fujita, Saki Noguchi, Rika Hamada, Satoko Inoue, Tsutomu Shimada, Satomi Katakura, Tetsuo Maruyama, Yoshimichi Sai, Tomohiro Nishimura, Masatoshi Tomi

**Affiliations:** 1 Division of Pharmaceutics, Faculty of Pharmacy, Keio University, 1-5-30 Shibakoen, Minato-ku, Tokyo, 105-8512 Japan; 2 Department of Clinical Pharmacokinetics, Graduate School of Medical Sciences, Kanazawa University, Kanazawa, Ishikawa 920-8641 Japan; 3 Department of Hospital Pharmacy, University Hospital, Kanazawa University, Kanazawa, Ishikawa 920-8641 Japan; 4 Department of Obstetrics and Gynecology, Keio University School of Medicine, Shinjuku-ku, Tokyo, 160-8582 Japan

**Keywords:** multidrug resistance protein 1 (MDR1), pharmacokinetic model, placenta, pregnancy, syncytiotrophoblasts

## Abstract

**Purpose:**

Multidrug resistance protein 1 (MDR1) is located at the interface between two syncytiotrophoblast layers in rodent placenta, and may influence fetal drug distribution. Here, we quantitatively compare the functional impact per single MDR1 molecule of MDR1 at the placental barrier and blood-brain barrier in mice.

**Methods:**

MDR1A and MDR1B proteins were quantified by liquid chromatography-tandem mass spectrometry (LC-MS/MS). Paclitaxel or digoxin was continuously administered to pregnant *Mdr1a*^−/−^/*Mdr1b*^−/−^ or wild-type mice, and the drug concentrations in the maternal and fetal plasma and maternal brain were quantified by LC-MS/MS.

**Results:**

MDR1A and MDR1B proteins are expressed in the membrane of mouse placental labyrinth, and total MDR1 at the placental barrier amounts to about 30% of that at the blood-brain barrier. The fetal-to-maternal plasma concentration ratio of digoxin was only marginally affected in *Mdr1a*^−/−^/*Mdr1b*^−/−^ mice, while that of paclitaxel showed a several-fold increase. No such difference between the two drugs was found in the maternal brain distribution. The impact per single MDR1 molecule on the fetal distribution of digoxin was calculated to be much lower than that on the brain distribution, but this was not the case for paclitaxel. Our pharmacokinetic model indicates that the impact of placental MDR1 is inversely correlated to the ratio of permeability through gap junctions connecting the two syncytiotrophoblast layers to passive diffusion permeability.

**Conclusion:**

Our findings indicate that murine placental MDR1 has a minimal influence on the fetal concentration of certain substrates, such as digoxin, due to bypass transfer, probably via connexin26 gap junctions.

**Supplementary Information:**

The online version contains supplementary material available at 10.1007/s11095-022-03165-6.

## Introduction

Multidrug resistance protein 1 (MDR1)/P-glycoprotein/ABCB1 is expressed in placental syncytiotrophoblasts (SynT) at the placental barrier and brain capillary endothelial cells at the blood-brain barrier, where it contributes to restricting drug distribution to the fetus and brain, respectively, by pumping its substrate drugs out to the systemic circulation. It is believed that the contribution of MDR1 to drug distribution depends primarily on the protein expression amount and functional activity of MDR1 protein at these barriers.

Indeed, human MDR1 protein in the placenta and brain is localized at the systemic circulation interface: the apical microvillous membrane (MVM) of a multinucleated SynT monolayer (1) and the luminal membrane of capillary endothelial cells (2), respectively. Rodent brain capillary endothelial cells also express MDR1 protein at the luminal membrane (3); however, we have demonstrated that the rodent placental MDR1 protein is not in contact with the systemic circulation (4). Namely, rodent SynT consists of a bilayer, with a maternal-facing layer I (SynT-I) and a fetal-facing layer II (SynT-II), and MDR1 proteins are localized at the apical membrane of the SynT-II layer. The basal plasma membrane of SynT-I and the apical membrane of SynT-II are connected by connexin26 gap junctions, (5, 6) which serve as intercellular channels and allow cells to exchange substances (7). Therefore, the ability of MDR1 to suppress fetal drug transfer may be reduced in rodents, because drugs can cross the barrier from the mother to fetus through the connexin26 gap junctions, bypassing MDR1. For example, the distribution of norbuprenorphine was increased in the maternal brain (~30-fold), but not in the fetus of pregnant *Mdr1a*^−/−^/*Mdr1b*^−/−^ (*Mdr1a/b*^−/−^) mice (8) (rodents have two MDR1 isoforms encoded by *Mdr1a*/*Abcb1a* and *Mdr1b*/*Abcb1b* genes), even though increased fetal distributions of digoxin, paclitaxel, and saquinavir have been reported in *Mdr1a/b*^*−/−*^ mice (9). These findings suggest that MDR1 at the placenta reduces fetal exposure to only some of its substrate drugs. It seems unlikely that this can be explained in terms of the MDR1 protein expression level.

The unbound plasma concentration ratios of fetal plasma to maternal plasma (*K*_p,uu,fm_) and brain to plasma (*K*_p,uu,brain_) at the steady state are the best measures of the extent of drug distribution to the fetus and the brain (10), respectively. The *K*_p,uu,fm_ and *K*_p,uu,brain_ of drugs that are not significantly metabolized in the fetus/placenta and the brain, respectively, are equivalent to the intrinsic clearance ratios of mother-to-fetus (*CL*_mf,int,all_) to fetus-to-mother (*CL*_fm,int,all_) and plasma-to-brain to brain-to-plasma, respectively. MDR1 decreases the intrinsic clearance ratio and *K*_p,uu_ of substrate drugs at the placenta and brain. It is reported that the *K*_p,uu,brain_ of MDR1 substrate drugs can be reconstructed from the *in vitro* MDR1 efflux ratio, which is the basal-to-apical/apical-to-basal transport ratio in a mouse *Mdr1a*-overexpressing LLC-PK1 cell monolayer divided by that in the parental LLC-PK1 cells, and the amounts of MDR1 protein in the brain capillaries and *Mdr1a*-overexpressing LLC-PK1 cells (11). The success of this reconstruction from *in vitro* to *in vivo* brain means that the decrement of the intrinsic clearance ratio due to a single MDR1 molecule in the *in vivo* brain is quantitatively almost the same as that *in vitro*. The decrement of the intrinsic clearance ratio due to a single MDR1 molecule at the placenta is also expected to be the same as that at the brain, but would be apparently diminished if transfer other than by MDR1, perhaps via connexin26 expressed in rodent placenta, significantly affects the drug distribution.

The embryo-fetal developmental toxicity of drugs has generally been estimated by the extrapolation of experimental findings in pregnant rodents. Therefore, it is important to quantify the effect of the spatial difference of MDR1 in rodent placenta on fetal drug distribution. In this study, we aimed to quantitatively compare the apparent contribution of a single MDR1 molecule to drug distribution across the placental barrier with that across the blood-brain barrier, using paclitaxel and digoxin as typical MDR1 substrates. Paclitaxel and digoxin have similar molecular weights of 853.93 and 780.96, respectively, but differ in lipophilicity, with log D at pH 7.4 of 6.83 and 1.26, respectively (12).

## Materials and Methods

### Preparation of Human Placental MVM-Enriched Fraction

Uncomplicated term human placental tissues were obtained with written informed consent, and with the approval of the Institutional Ethics Committee of Keio University Faculty of Pharmacy (150421–2) and Keio University School of Medicine (20110250). Human placentas were collected from pregnant women who had undergone elective caesarean section due to previous caesarean section or breech presentation. Human placental MVM-enriched fraction was prepared by magnesium precipitation as previously reported (13). The activity of alkaline phosphatase, an MVM marker, in human MVMs was enriched 11.4 ± 0.23-fold (*n* = 4) relative to those in the villous homogenate, respectively.

### Animals

*Mdr1a/b*^*−/−*^ FVB mice were purchased from Taconic (Hudson, NY). The wild-type(WT) FVB mice were purchased from CLEA Japan (Tokyo, Japan). Mice were maintained under a 12 h/12 hlight-dark cycle at 25°C with free access to water and food until use. Female WT and *Mdr1a/b*^*−/−*^ mice were mated with male mice of the same genotype. The presence of a vaginal plug was designated as gestational day (GD) 0.5. Animal experiments were approved by the Institutional Animal Care Committee and complied with the standards set out in the Guideline for the Care and Use of Laboratory Animals in Keio University.

### Preparation of Plasma Membrane Fraction from Mouse Placental Labyrinth

Plasma membrane fraction was prepared from mouse placental labyrinth as described previously (14). Placentas were isolated from GD13.5, 15.5, and 17.5 pregnant WT and GD17.5 *Mdr1a/b*^*−/−*^ mice. The labyrinth was collected from the isolated placentas using tweezers and homogenized in Tris-sucrose buffer (250 mM sucrose, 10 mM Tris/HCl, 1 μM pepstatin A, 10 μM leupeptin, 100 μM phenylmethanesulfonyl fluoride, pH 7.4). The homogenate was centrifuged at 5800 g for 15 min at 4°C, and the supernatant was centrifuged at 10,000 g for 15 min at 4°C. The resulting supernatant was ultracentrifuged at 124,000 g for 30 min at 4°C. The pellet was suspended in Tris-sucrose buffer, layered on top of 38% (wt/vol) sucrose solution, and centrifuged at 10,000 g for 40 min at 4°C with a swing-out rotor. The turbid layer at the interface was recovered, suspended in 10 mM Tris-HCl buffer (pH 7.4), and centrifuged at 10,000 g for 40 min at 4°C. The resultant pellet was resuspended in Tris-sucrose buffer and used as the plasma membrane fraction. Protein concentration was measured by the Bradford method using Protein Assay reagent (Bio-Rad, Hercules, CA, USA) with bovine serum albumin as a standard. The plasma membrane fraction was stored at −80°C until use.

### LC-MS/MS-Based Targeted Protein Quantification Analysis

The expression amounts of transporter proteins were measured by quantifying the absolute amounts of specific (ST) peptides generated from the target transporter proteins by means of LC-MS/MS, as described previously (15, 16). The peptide sequences for human MDR1 (NP_001335874.1) and mouse MDR1A (NP_035206.2) proteins are NTTGAL*TTR (15), and that for MDR1B (NP_035205.1) protein is TVIAFGGQQK* (17). The asterisk (*) indicates the amino acid labeled with ^13^C and ^15^N in the internal standard (IS) peptides. Briefly, the plasma membrane fraction (100 μg protein) was reduced and alkylated. The alkylated proteins were precipitated with methanol and chloroform for purification. The precipitated proteins were dissolved in urea and treated first with lysyl endopeptidase and ProteaseMAX surfactant (Promega, Madison, WI, USA) and then with *N*-tosyl-L-phenylalanine chloromethyl ketone-treated trypsin (Promega). The resulting peptide samples were spiked with IS peptides and acidified with formic acid.

The LC-MS/MS system consisted of a high-performance liquid chromatography instrument and an electrospray ionization triple quadrupole mass spectrometer (LCMS-8050; Shimadzu, Kyoto, Japan) operated in the positive ionization mode. Samples were injected into an XBridge BEH130 C18 column (1 mm I.D. × 100 mm, 3.5 μm, Waters, Milford, MA) on the LC system at 40°C and eluted with a linear gradient of acetonitrile containing 0.1% formic acid. The eluted peptides were quantified by multiplexed selected reaction monitoring (SRM) using LabSolutions software (Shimadzu). The peak data were extracted by using 3 sets of SRM transitions (m/z) of the precursor and product ions (Q1/Q3: 467.8/719.4, 467.8/216.1, and 467.8/561.3 for human MDR1 ST and mouse MDR1A ST, 471.3/726.4, 471.3/216.1, and 471.3/568.3 for MDR1 and MDR1A IS, 524.8/848.5, 524.8/735.4, and 524.8/517.3 for mouse MDR1B ST, and 528.8/856.5, 528.8/743.4, and 528.8/525.3 for MDR1B IS) per peptide with the dwell time of 10 msec per transition. A peak was defined as positive when the signal-to-noise ratio was over 3. The amount of the peptide in the sample was first determined for each transition using the peak area ratio (ST/IS) of the positive peak and a calibration curve obtained with known concentrations of synthetic peptide, and was subsequently expressed as the average of 3 positive peaks from different transitions, as presented in Supplemental Table S1.

### Administration of Drugs to Pregnant Mice

Paclitaxel or digoxin was continuously administered to pregnant *Mdr1a/b*^*−/−*^ or WT mice from GD13.5 and 15.5 for 48 h (paclitaxel) or from GD12.5 and 14.5 for 72 h (digoxin) using an osmotic pump (Alzet model 1003D: Durect, Cupertino, CA, USA) with a pumping rate of 1.0 μL/h. Osmotic pumps were filled with paclitaxel (Taxol®, Bristol Myers Squibb) diluted to 3.51 mM with water or digoxin (230 μM) dissolved in 80% PEG400/DMSO and implanted into the subcutaneous tissue of the backs of mice under deep anesthesia with isoflurane. Maternal blood was collected from a tail vein in studies to determine the maternal plasma concentration during the continuous administration. At 48 or 72 h after starting administration, the fetuses were decapitated for collection of fetal blood with a heparinized microhematocrit capillary tube. The maternal blood was collected from the left jugular vein, and then the cerebrum of the mother was excised and weighed.

### Measurement of Drug Concentrations in Plasma and Tissues

Blood was centrifuged at 15,000 rpm for 20 min at 4°C to obtain plasma. The cerebrum was homogenized with water (paclitaxel-administered samples) or 10 mM ammonium acetate (digoxin-administered samples) to obtain 20% homogenate. For paclitaxel determination, the plasma and brain homogenate were diluted with 5 volumes of tert-butyl methyl ether and spiked with docetaxel as an IS. For digoxin determination, the plasma and brain homogenate were diluted with two volumes of acetonitrile containing 0.1% formic acid and spiked with digitoxin as an IS. After vortexing, the samples were centrifuged at 15,000 g for 10 min at 4°C. The supernatant was evaporated in a vacuum centrifuge. The residue was reconstituted in 50% (paclitaxel) or 20% (digoxin) acetonitrile containing 0.1% formic acid and centrifuged at 15,000 g for 1 min at 4°C.

Supernatant samples were used for drug quantification by LC-MS/MS. HPLC separation was performed on a Capcell Pak C18 UG120 column (2.0 mm I.D. × 150 mm, 5 μm, Osaka Soda, Osaka, Japan) for paclitaxel determination or a Shim-pack GISS C18 UG120 (2.1 mm I.D. × 100 mm, 1.9 μm, Shimadzu) for digoxin determination at 40°C with a gradient of mobile phases A and B consisting of 0.1% formic acid in water and 0.1% formic acid in acetonitrile, respectively. The gradient of mobile phase B was as follows: 10% for 2 min (at 0–2 min), 10–100% for 4 min (at 2–6 min), 100% for 3 min (at 6–9 min), and 10% for 4 min (at 9–13 min) at a flow rate of 0.6 mL/min for paclitaxel determination, and 20% for 2 min (at 0–2 min), 20–100% for 2 min (at 2–4 min), 100% for 2 min (at 4–6 min), and 20% for 2 min (at 6–8 min) at a flow rate of 0.4 mL/min for digoxin determination. The eluted drugs were quantified by SRM, with the mass spectrometer operating in the positive ionization mode, using m/z of Q1/Q3: 876.0/308.0 for paclitaxel, 830.0/549.1 for docetaxel (IS for paclitaxel determination), 779.4/649.25 for digoxin, and 763.4/503.4 for digitoxin (IS for digoxin determination). The analyte concentration was determined from the peak area ratio to the internal standard by the use of a calibration curve. The lower limits of quantification for paclitaxel and digoxin were 0.2 ng/mL and 0.5 ng/mL, respectively.

### Determination of Unbound Fraction of Drugs

The unbound fraction was determined by equilibrium dialysis using a rapid equilibrium dialysis device (Thermo Fisher Scientific, Waltham, MA, USA). Paclitaxel or [^3^H(G)]digoxin ([^3^H] digoxin, 39.8 Ci/mmol; PerkinElmer, Boston, MA, USA) was spiked into maternal or fetal plasma at a final concentration of 26 or 13 nM. Spiked plasma (100 μL) was loaded into the sample chambers of the device, and the buffer chambers were filled with 350 μL of phosphate-buffered saline. The device was covered with sealing tape and incubated for 12 h at 37°C on an orbital shaker running at 250 rpm. Following incubation, aliquots from both chambers were taken for measurement of paclitaxel concentration or radioactivity of [^3^H]digoxin using LC-MS/MS or liquid scintillation counting, respectively. The unbound fraction in the maternal and fetal plasma (*f*_u,mp_ and *f*_u,fp_) was calculated as the PBS-to-plasma concentration (radioactivity) ratio. Albumin concentration in maternal and fetal plasma was determined using an LBIS Mouse Albumin ELISA Kit (Fujifilm Wako Shibayagi, Shibukawa, Japan) according to the manufacturer’s protocol.

### Determination of Fetal and Brain Distribution of Drugs

The ratio of fetal-to-maternal plasma concentration ratio (*K*_p,fm_) was calculated by dividing fetal plasma concentration by maternal plasma concentration at the steady state. Since the transfer rate from maternal plasma to fetal plasma and the sum of the transfer rate from fetal plasma to maternal plasma and the fetal metabolic rate are in equilibrium at the steady state, *K*_p,fm_ can be replaced with the ratio of the plasmatic clearance in the maternal-to-fetal direction (*CL*_mf_) to the sum of the plasmatic clearance in the fetal-to-maternal direction (*CL*_fm_) and the fetal metabolic clearances. However, fetal metabolism is assumed to be insignificant for the drugs used in this study, digoxin and paclitaxel, for the following reasons: digoxin is predominantly eliminated through the maternal kidney and not extensively metabolized in the body. The systemic elimination of paclitaxel occurs in humans by hepatic metabolism involving CYP3A4 and CYP2C8, and that in FVB mice most likely occurs via similar mechanisms (18). The expression levels of most CYP enzymes, including murine orthologs of human CYP3A4 and CYP2C8, in mouse fetal liver are very low except for CYP3A16 (murine ortholog of human CYP3A7) (19). The catalysis of 4-hydroxylation of retinoic acid by CYP3A7 in human fetal liver microsomes is not affected by paclitaxel (20).

*CL*_mf_ and *CL*_fm_ of drugs at the steady state are equivalent to the product of *f*_u,mp_ and *CL*_mf,int,all_ and the product of *f*_u,fp_ and *CL*_fm,int,all_ respectively. Accordingly, *K*_p,fm_ and *K*_p,uu,fm_ are given by

1 $$ {K}_{\mathrm{p},\mathrm{fm}}=\frac{f_{\mathrm{u},\mathrm{mp}}\times {CL}_{\mathrm{mf},\operatorname{int},\mathrm{all}}}{f_{\mathrm{u},\mathrm{fp}}\times {CL}_{\mathrm{fm},\operatorname{int},\mathrm{all}}} $$

2 $$ {K}_{\mathrm{p},\mathrm{uu},\mathrm{fm}}={K}_{\mathrm{p},\mathrm{fm}}\times \frac{f_{\mathrm{u},\mathrm{fp}}}{f_{\mathrm{u},\mathrm{mp}}}=\frac{CL_{\mathrm{mf},\operatorname{int},\mathrm{all}}}{CL_{\mathrm{fm},\operatorname{int},\mathrm{all}}} $$

**Fig. 1:**
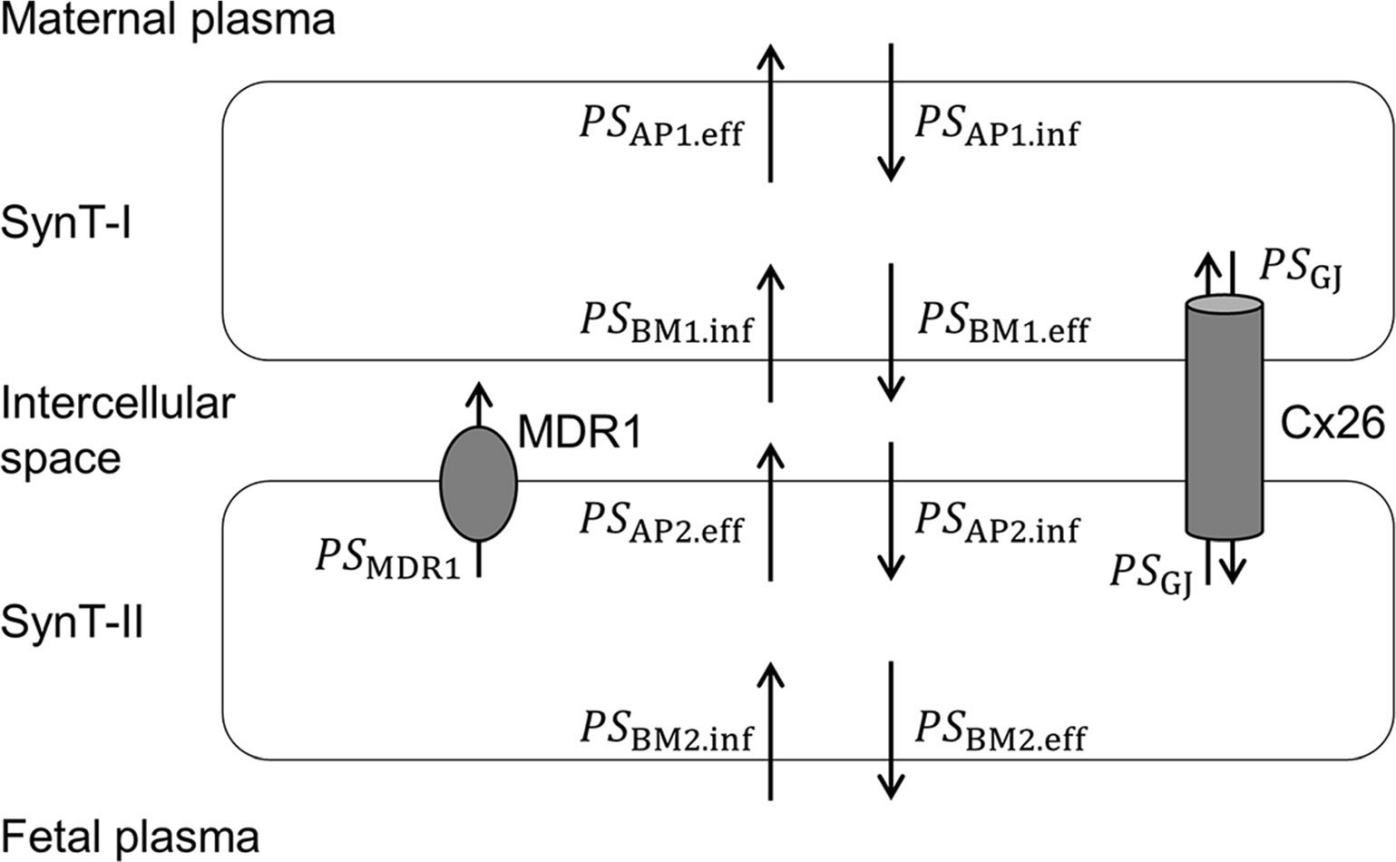
Pharmacokinetic model illustrating the PS products for transplacental transfer of MDR1 substrates in rodents.

In rodents, the SynT bilayer forming the placental barrier consists of a maternal-facing layer I (SynT-I) and a fetal-facing layer II (SynT-II) connected at frequent intervals by the gap-junctional protein connexin26 (5–7), and MDR1 is localized at the apical membrane of SynT-II(4). Accordingly, we can develop a transplacental pharmacokinetic model for MDR1 substrates as illustrated in Fig. 1. *CL*_mf,int,all_ and *CL*_fm,int,all_ are hybrid parameters as indicated below, including uptake and efflux at the apical and basal plasma membranes of SynT-I and SynT-II and the intercellular transfer between SynT-I and SynT-II through gap junctions (see Supplemental Text for details).

3 $$ {CL}_{\mathrm{mf},\operatorname{int},\mathrm{all}}={PS}_{\mathrm{AP}1.\operatorname{inf}}\times \frac{\beta_{12}\times {PS}_{\mathrm{BM}2.\mathrm{eff}}}{\beta_{12}\times {PS}_{\mathrm{BM}2.\mathrm{eff}}+{\beta}_{21}\times {PS}_{\mathrm{AP}1.\mathrm{eff}}+{PS}_{\mathrm{BM}2.\mathrm{eff}}\times {PS}_{\mathrm{AP}1.\mathrm{eff}}} $$

4 $$ {CL}_{\mathrm{fm},\operatorname{int},\mathrm{all}}={PS}_{\mathrm{BM}2.\operatorname{inf}}\times \frac{\beta_{21}\times {PS}_{\mathrm{AP}1.\mathrm{eff}}}{\beta_{12}\times {PS}_{\mathrm{BM}2.\mathrm{eff}}+{\beta}_{21}\times {PS}_{\mathrm{AP}1.\mathrm{eff}}+{PS}_{\mathrm{BM}2.\mathrm{eff}}\times {PS}_{\mathrm{AP}1.\mathrm{eff}}} $$

5 $$ {\beta}_{12}={PS}_{\mathrm{BM}1.\mathrm{eff}}\times \frac{P{S}_{\mathrm{AP}2.\operatorname{inf}}}{P{S}_{\mathrm{BM}1.\operatorname{inf}}+{PS}_{\mathrm{AP}2.\operatorname{inf}}}+{PS}_{\mathrm{GJ}} $$

6 $$ {\beta}_{21}=\left({PS}_{\mathrm{MDR}1,\mathrm{PB}}+{PS}_{\mathrm{AP}2.\mathrm{eff}}\right)\times \frac{P{S}_{\mathrm{BM}1.\operatorname{inf}}}{P{S}_{\mathrm{BM}1.\operatorname{inf}}+{PS}_{\mathrm{AP}2.\operatorname{inf}}}+{PS}_{\mathrm{GJ}} $$

where *PS*_AP1.inf_ and *PS*_AP1.eff_ represent the permeability-surface area (PS) product for influx and efflux across the apical membrane of SynT-I, respectively; *PS*_BM1.inf_ and *PS*_BM1.eff_ represent the PS product for influx and efflux across the basal plasma membrane of SynT-I, respectively; *PS*_AP2.inf_ and *PS*_AP2.eff_ represent the PS product for influx and efflux across the apical membrane of SynT-II, excluding MDR1-mediated efflux, respectively; *PS*_BM2.inf_ and *PS*_BM2.eff_ represent the PS product for influx and efflux across the basal plasma membrane of SynT-II, respectively; *PS*_MDR1,PB_ represents the PS product for efflux mediated by MDR1 at the placental barrier, and *PS*_GJ_ represents the PS product for gap junction-mediated transfer between SynT-I and SynT-II. Accordingly, *K*_p,fm_ is given by

7 $$ {K}_{\mathrm{p},\mathrm{fm}}=\frac{f_{\mathrm{u},\mathrm{mp}}}{f_{\mathrm{u},\mathrm{fp}}}\times \frac{P{S}_{\mathrm{AP}1.\operatorname{inf}}}{P{S}_{\mathrm{AP}1.\mathrm{eff}}}\times \frac{\beta_{12}}{\beta_{21}}\times \frac{P{S}_{\mathrm{BM}2.\mathrm{eff}}}{P{S}_{\mathrm{BM}2.\operatorname{inf}}} $$

As a parameter describing the rodent placental MDR1 function, the *K*_p,fm_ ratio is obtained from *K*_p,fm_ in WT and *Mdr1a/b*^*−/−*^ mice as follows:

8 $$ {K}_{\mathrm{p},\mathrm{fm}}\mathrm{ratio}=\frac{K_{\mathrm{p},\mathrm{fm},\mathrm{KO}}}{K_{\mathrm{p},\mathrm{fm},\mathrm{WT}}}=\frac{\beta_{21,\mathrm{WT}}}{\beta_{21,\mathrm{KO}}}=\frac{\left({PS}_{\mathrm{MDR}1,\mathrm{PB}}+{PS}_{\mathrm{AP}2.\mathrm{eff}}\right)\times \frac{P{S}_{\mathrm{BM}1.\operatorname{inf}}}{P{S}_{\mathrm{BM}1.\operatorname{inf}}+{PS}_{\mathrm{AP}2.\operatorname{inf}}}+{PS}_{\mathrm{GJ}}}{P{S}_{\mathrm{AP}2.\mathrm{eff}}\times \frac{P{S}_{\mathrm{BM}1.\operatorname{inf}}}{P{S}_{\mathrm{BM}1.\operatorname{inf}}+{PS}_{\mathrm{AP}2.\operatorname{inf}}}+{PS}_{\mathrm{GJ}}}\kern1.25em =1+\frac{P{S}_{\mathrm{MDR}1,\mathrm{PB}}}{P{S}_{\mathrm{AP}2.\mathrm{eff}}+{PS}_{\mathrm{GJ}}\times \left(1+\frac{P{S}_{\mathrm{AP}2.\operatorname{inf}}}{P{S}_{\mathrm{BM}1.\operatorname{inf}}}\right)} $$

### Comparison of the Contributions of MDR1 to Drug Distribution in the Placental and the Blood-Brain Barriers

The mouse placental barrier expresses MDR1A encoded by *Mdr1a* and *Mdr1b* genes, (21–23) while the blood-brain barrier only expresses MDR1A (17, 24). It is reported that the efflux ratios of 12 drugs including digoxin and paclitaxel, calculated by dividing the PS product for the basal-to-apical direction by that for the apical-to-basal direction, correlate well between MDR1A- and MDR1B-stably expressing LLC-PK1 cells (25). Therefore, it is assumed here that MDR1A and MDR1B are functionally equivalent.

The maternal brain-to-plasma concentration ratio (*K*_p,brain_) in WT and *Mdr1a/b*^*−/−*^ mice was used for calculating the *K*_p,brain_ ratio as a parameter describing MDR1 function at the blood-brain barrier according to previous reports (11, 26).

9 $$ {K}_{\mathrm{p},\mathrm{brain}}\ \mathrm{ratio}=\frac{K_{\mathrm{p},\mathrm{brain},\mathrm{KO}}}{K_{\mathrm{p},\mathrm{brain},\mathrm{WT}}}=1+\frac{PS_{\mathrm{MDR}1,\mathrm{BBB}}}{PS_{\mathrm{l}.\mathrm{eff}}} $$

where *PS*_MDR1,BBB_ and *PS*_l.eff_ represent the PS product for efflux mediated by MDR1 and for luminal efflux excluding MDR1-mediated efflux, respectively, at brain capillary endothelial cells.

It has been reported that MDR1 transport activity is approximately proportional to MDR1 protein expression amount (27, 28). Accordingly, the MDR1 contribution to fetal drug distribution, expressed as *K*_p,fm_ ratio − 1, per single MDR1 molecule at the placental barrier can be obtained from that at the blood-brain barrier by using a conversion ratio (*R*_P/B_), given by

10 $$ \kern0.75em {R}_{\mathrm{P}/\mathrm{B}}=\frac{\frac{P{S}_{\mathrm{MDR}1,\mathrm{PB}}}{P{S}_{\mathrm{AP}2.\mathrm{eff}}+{PS}_{\mathrm{GJ}}\times \left(1+\frac{P{S}_{\mathrm{AP}2.\operatorname{inf}}}{P{S}_{\mathrm{BM}1.\operatorname{inf}}}\right)}\times \frac{1}{\begin{array}{c}\mathrm{MDR}1\mathrm{proteinamounts}\\ {}\mathrm{inapicalsideofSynT}-\mathrm{II}\left(\mathrm{fmol}/\upmu \mathrm{gprotein}\right)\end{array}}}{\frac{P{S}_{\mathrm{MDR}1,\mathrm{BBB}}}{P{S}_{\mathrm{l}.\mathrm{eff}}}\times \frac{1}{\begin{array}{c}\mathrm{MDR}1\mathrm{proteinamounts}\\ {}\mathrm{inluminalsideofbraincapillaries}\left(\mathrm{fmol}/\upmu \mathrm{gprotein}\right)\end{array}}}=\frac{P_{\mathrm{l}.\mathrm{eff}}}{P_{\mathrm{AP}2.\mathrm{eff}}+{P}_{\mathrm{GJ}}\times \left(1+\frac{P_{\mathrm{AP}2.\operatorname{inf}}}{P_{\mathrm{BM}1.\operatorname{inf}}}\right)} $$

where *P*_l.eff_ represents the luminal efflux permeability excluding MDR1-mediated efflux at brain capillary endothelial cells; *P*_AP2.inf_ and *P*_AP2.eff_ represent the permeability for influx and efflux, respectively, across the apical membrane of SynT-II, excluding MDR1-mediated efflux; *P*_BM1.inf_ represents the influx permeability across the basal plasma membrane of SynT-I; and *P*_GJ_ represents the permeability for transfer mediated by gap junctions.

### Data Analysis

Statistical analyses were performed using an unpaired, 2-tailed Student’s *t* test. The value of *p* < 0.05 was taken as the criterion for a statistically significant difference.

All data represent the mean ± S.E.M. unless otherwise indicated. The S.E.M. was calculated according to the following law of propagation of errors, given the following functional relationship between several measured variables such as y, x_1_, x_2_, ………, x_n_ (for example, in Eq. 8, y can be the *K*_p,fm_ ratio, and x_1_ and x_2_ can be *K*_p,fm_ in *Mdr1a/b*^*−/−*^ or WT mice)

11 $$ \mathrm{y}=f\left({\mathrm{x}}_1,{\mathrm{x}}_2,\dots \dots, {\mathrm{x}}_{\mathrm{n}}\right) $$

If the variables x_1_, x_2_, ………, x_n_ are uncorrelated, the standard deviations of y (S. D._y_) and (S. D._x_) are related as follows:

12 $$ \mathrm{S}.\mathrm{D}{._{\mathrm{y}}}^2={\left(\frac{\mathrm{\partial f}}{{\mathrm{\partial x}}_1}\right)}^2\mathrm{S}.\mathrm{D}{._{{\mathrm{x}}_1}}^2+{\left(\frac{\mathrm{\partial f}}{{\mathrm{\partial x}}_2}\right)}^2\mathrm{S}.\mathrm{D}{._{{\mathrm{x}}_2}}^2+\cdots \cdots +{\left(\frac{\mathrm{\partial f}}{{\mathrm{\partial x}}_{\mathrm{n}}}\right)}^2\mathrm{S}.\mathrm{D}{._{{\mathrm{x}}_{\mathrm{n}}}}^2 $$

where $\frac{\mathrm{\partial f}}{\mathrm{\partial x}}$ is the partial derivative of function y concerning x.

The S.E.M. of y is given by

13 $$ \mathrm{S}.\mathrm{E}.\mathrm{M}.=\frac{\mathrm{S}.\mathrm{D}{.}_{\mathrm{y}}}{\sqrt{\mathrm{n}\left(\mathrm{y}\right)}} $$

where n(y) is assumed to be n(y) = n(x_1_) × n(x_2_) × ⋯⋯ × n(x_n_).

## Results

### Absolute Protein Amounts of MDR1 in Placenta During Gestation

**Fig. 2:**
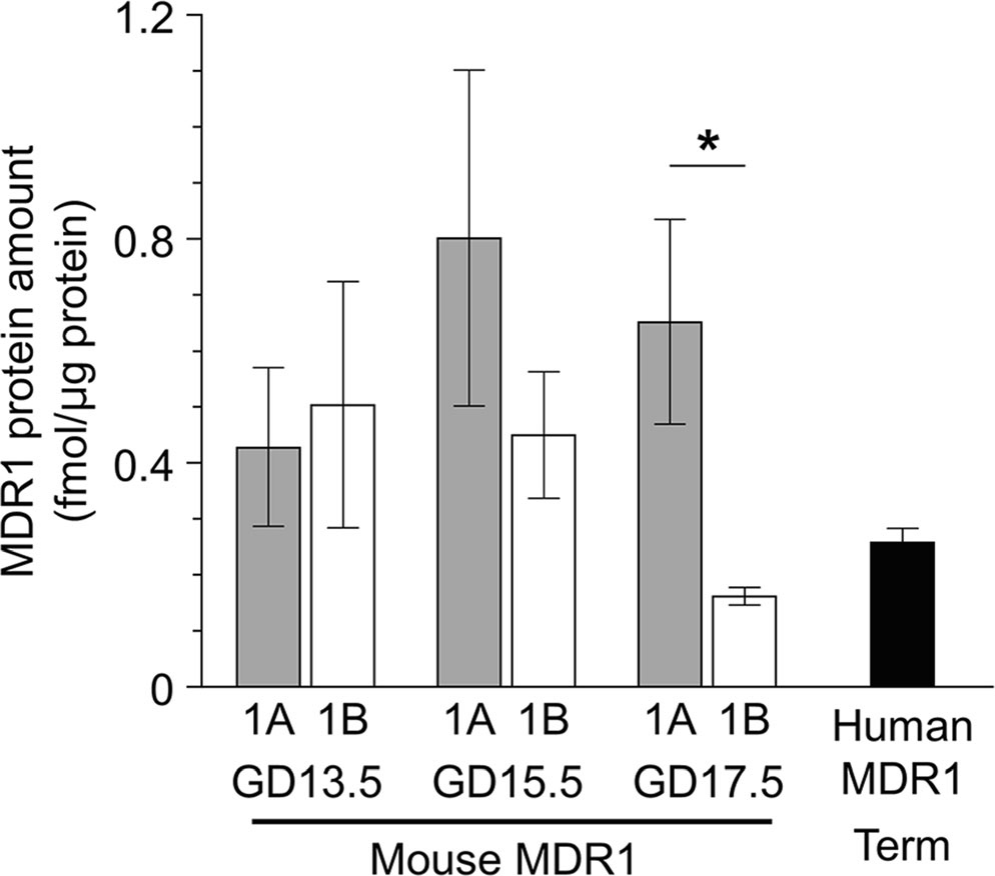
MDR1 protein amounts in the placenta. The absolute protein expression amounts of mouse MDR1A and MDR1B in plasma membrane fraction of the mouse placental labyrinth and human MDR1 in human placental microvillous membrane-enriched fraction. Each column represents the mean ± S.E.M. of 4 human donors or 3 independent sample preparations from mouse placenta. The peak data of the peptides were extracted by using 3 sets of SRM transitions and the expression levels of individual donors or samples were determined as the mean of 3 quantitative data. The quantitative data of each SRM transition of individual donors or samples are shown in Supplemental Table S1. *p < 0.05, significant difference between MDR1A and MDR1B at the same gestational day.

Two mouse MDR1 protein isoforms, MDR1A and MDR1B, were expressed in plasma membrane fractions of the mouse placental labyrinth at GD13.5, 15.5, and 17.5. Positive peaks at the same retention time as the IS peptides were observed in chromatograms of three SRM transitions for MDR1A and MDR1B in WT placenta, but not in *Mdr1a/b*^*−/−*^ placenta (Supplemental Fig. S1), indicating the expression of MDR1A and MDR1B proteins in the plasma membrane fraction of WT placental labyrinth. Protein expression amounts of MDR1A and MDR1B were similar (within the range of 0.43 to 0.80 fmol/μg protein) at GD13.5 and 15.5 (Fig. 2). However, MDR1B expression at GD17.5, a day before labor starts in FVB mice (29), fell to 0.16 fmol/μg protein, which was the lowest among the three gestational days and significantly lower than the MDR1A expression amount at GD17.5 (0.65 fmol/μg protein). The amount of MDR1 proteins at the apical membrane of SynT-II cells and the luminal membrane of mouse brain capillary endothelial cells were estimated and compared according to the calculation shown in Supplemental Text. The MDR1 protein amount at the apical membrane of SynT-II cells is estimated to be 23–35% of that at the luminal membrane of mouse brain capillary endothelial cells (15) (Table I).

**Table I:** Estimated Expression Amounts of MDR1 in Mouse Placental Barrier and Blood-Brain Barrier

	GD	MDR1 Protein (fmol/μg protein)
Apical membrane of placental SynT-II	15.5	10
	17.5	7.0
Luminal membrane of brain capillaries	–	28

The expression amount of MDR1 at the apical membrane of placenta SynT-II was estimated to be 8 times larger than that in plasma membrane fraction of the placental labyrinth (Fig. 2). The expression amount of MDR1 at the luminal membrane of brain capillaries was estimated to be twice that in plasma membrane fraction of brain capillaries reported by Uchida *et al*. (2011)(11)(See Supplemental Text for details)

The amount of MDR1 protein in the human placental MVM-enriched fraction was determined to be 0.26 fmol/μg protein (Fig. 2), which is 4.0% of the estimated MDR1 protein expression amount at the apical membrane of SynT-II cells in pregnant mice at GD17.5.

### Effect of MDR1 on Fetal and Brain Distributions of Paclitaxel and Digoxin

The fetal plasma (fp) and maternal brain distributions of paclitaxel and digoxin were compared between pregnant WT and *Mdr1a/b*^*−/−*^ mice at GD15.5 and 17.5 after continuous administration for 48 and 72 h, respectively. Maternal and fetal plasma concentrations in pregnant WT mice are shown in Supplemental Fig. S2. As shown in Table II, *Mdr1a/b*^*−/−*^ mice exhibited a several-fold increase in *K*_p,fm_ of paclitaxel compared with WT mice, while the *K*_p,fm_ ratio (KO/WT) of digoxin was 1.4. On the other hand, MDR1 clearly affected the brain distribution of paclitaxel and digoxin, and the *K*_p,brain_ ratio of digoxin is even higher than that of paclitaxel. The higher *K*_p,brain_ ratio of digoxin than paclitaxel appears to be in good agreement with the intrinsic nature of MDR1 transport, since the *in vitro* MDR1 efflux ratio of digoxin, which is the basal-to-apical/apical-to-basal transport ratio in a monolayer of cells apically expressing MDR1 divided by that in non-expressing cells, is reported to be consistently higher than that of paclitaxel (11, 25). The reported *in vitro* MDR1A efflux ratios of digoxin and paclitaxel determined using mouse MDR1A-transfected LLC-PK1 cells and parental cells (11) are summarized in Table II.

**Table II:** Distribution of Paclitaxel and Digoxin to Fetal Plasma and Brain in wild-Type(WT) and *Mdr1a*^−/−^/*1b*^−/−^(KO) Mice

	GD	*K*_p,fm WT_	*K*_p,fm KO_	*K*_p,fm_ ratio (KO/WT)	*K*_p,brain WT_	*K*_p,brain KO_	*K*_p,brain_ Ratio (KO/WT)	*In vitro* MDR1A Efflux Ratio^a^
Paclitaxel	15.5	0.0289 ± 0.0100	0.0922 ± 0.0112^*^	3.20 ± 0.68	0.443 ± 0.080	12.3 ± 1.4^**^	27.9 ± 2.30	12.3 ± 1.3
	17.5	0.0112 ± 0.0036	0.0837 ± 0.0094^**^	7.49 ± 1.48				
Digoxin	15.5	0.214 ± 0.014	0.298 ± 0.059	1.40 ± 0.17	0.348 ± 0.056	14.8 ± 3.0^**^	42.6 ± 3.27	17.8 ± 2.1
	17.5	0.216 ± 0.041	0.307 ± 0.052	1.42 ± 0.15				

The *K*_p_ value in fetal plasma and brain was measured after continuous administration of paclitaxel or digoxin at a rate of 1.0 μL/h for 48 or 72 h, respectively, using osmotic pumps filled with 3.51 mM paclitaxel or 230 μM digoxin. The plasma and brain were collected at GD15.5 and 17.5, and *K*_p,fm_ was determined according to the gestational day. *K*_p,brain_ was determined without distinction of the gestational day. Each value represents the mean ± S.E.M. (*n* = 3–9). The S.E.M. of the *K*_p,fm_ ratio was calculated according to the law of propagation of error. ^a^Data from Uchida *et al*. (2011)(11). ^*^p < 0.05, ^**^p < 0.01, significant difference from *K*_p_ in WT

The apparent impact of a single MDR1 molecule on the drug distribution can be calculated as the change in the *K*_p_ ratio (Table II), i.e., *K*_p_ ratio − 1, divided by the estimated MDR1 protein expression amount (Table I). Then, the ratio of the change of *K*_p,fm_ ratio per single placental MDR1 molecule to the change of *K*_p,brain_ ratio per single brain MDR1 molecule was determined as *R*_P/B_(Table III). Similarly, the ratio of the change of *K*_p,fm_ ratio per single placental MDR1 molecule to the change of *in vitro* MDR1A efflux ratio per single MDR1 molecule (Table II) (11) was also determined as *R*_P/C_ (Table III). *R*_P/B_ and *R*_P/C_ of paclitaxel were within the range of 0.23 to 2.5, suggesting that placental MDR1 can serve as an efflux pump for paclitaxel. On the other hand, *R*_P/B_ and *R*_P/C_ of digoxin were less than 0.11, suggesting that placental MDR1 does not work efficiently as an efflux pump for digoxin.

**Table III:** Placenta-to-Brain Ratio (*R*_P/B_) and Placenta-to-*In Vitro* MDR1A Cell Ratio (*R*_P/C_) of the Apparent Impact per Single MDR1 Protein Molecule on the Distribution of Paclitaxel and Digoxin

	GD	*R*_P/B_	*R*_P/C_
Paclitaxel	15.5	0.232 ± 0.002	0.551 ± 0.013
	17.5	1.05 ± 0.01	2.50 ± 0.06
Digoxin	15.5	0.0271 ± 0.0002	0.0673 ± 0.0021
	17.5	0.0443 ± 0.0003	0.1100 ± 0.0081

The *R*_P/B_ and *R*_P/C_ values were determined as the ratio of *K*_p,fm_ ratio − 1 per single placental MDR1 molecule to *K*_p,brain_ ratio − 1 per single brain MDR1 molecule and to the change of *in vitro* MDR1A efflux ratio per single MDR1 molecule, respectively. The protein expression amounts of MDR1 and *K*_p_ ratios are given in Tables I and II, respectively. Each value represents the mean ± S.E.M. The S.E.M. was calculated according to the law of propagation of error

A transplacental pharmacokinetic model of MDR1 substrates in mice (Fig. 1) was developed as described in theMaterials and Methods section to understand the effect of MDR1 on fetal drug distribution. According to Eq. (9), the change of *K*_p,brain_ ratio can be simply expressed as the PS product ratio of MDR1-mediated efflux (*PS*_MDR1,BBB_) to non-MDR1-mediated efflux (*PS*_l.eff_), and the change of *in vitro* MDR1A efflux ratio can also be expressed similarly (Supplemental Text). However, according to Eq. (8), the change of *K*_p,fm_ ratio can be expressed as the PS product ratio of MDR1-mediated efflux (*PS*_MDR1,PB_) to non-MDR1-mediated efflux (*PS*_AP2.eff_) only when the gap junction-mediated transfer (*PS*_GJ_) is negligible, otherwise other carrier(s) that increase *PS*_AP2.inf_and/or*PS*_GJ_, such as connexin26, reduce the change of *K*_p,fm_ ratio. Accordingly, it seems likely that *PS*_GJ_ of digoxin is sufficiently large to reduce the suppressive effect of MDR1 on the fetal distribution to less than 10%.

### Unbound Fractions of Paclitaxel and Digoxin

In order to determine the *K*_p,uu,fm_ values, the unbound fractions of paclitaxel and digoxin were measured in maternal (*f*_u,mp_) and fetal plasma (*f*_u,fp_) of WT mice. The *f*_u,fp_ values of paclitaxel and digoxin are all higher than the *f*_u,mp_ values, but the differences between *f*_u,fp_ and *f*_u,mp_ are smaller at GD17.5 than at GD15.5 (Table IV). The concentrations of albumin and paclitaxel in fetal plasma are much lower than those in maternal plasma, but increase with gestation (Table IV). Since digoxin binds to albumin (30) and paclitaxel binds to albumin and α-acid glycoprotein (31), the higher *f*_u,fp_ values especially at GD15.5 would be at least partly due to the low albumin concentration in the fetal plasma.

**Table IV:** Unbound Fraction of Paclitaxel and Digoxin in Maternal (*f*_u,mp_) and Fetal (*f*_u,fp_) Plasma and Albumin Concentration in Maternal and Fetal Plasma of Pregnant Mice

GD	Paclitaxel		Digoxin		Maternal albumin (mg/mL)	Fetal albumin (mg/mL)
	*f*_u,mp_	*f*_u,fp_	*f*_u,mp_	*f*_u,fp_		
15.5	0.0548 ± 0.0027	0.198 ± 0.003	0.428 ± 0.005	0.854 ± 0.001	44.1 ± 6.3	2.84 ± 0.44
17.5	0.0733 ± 0.0037	0.201 ± 0.015	0.650 ± 0.007	0.777 ± 0.012	39.1 ± 3.2	8.93 ± 0.81

The unbound fraction of paclitaxel (26 nM) and [^3^H]digoxin (13 nM) was determined by equilibrium dialysis. The albumin concentration in maternal and fetal plasma was measured by ELISA. Each value represents the mean ± S.E.M. (*n* = 3–5)

**Fig. 3:**
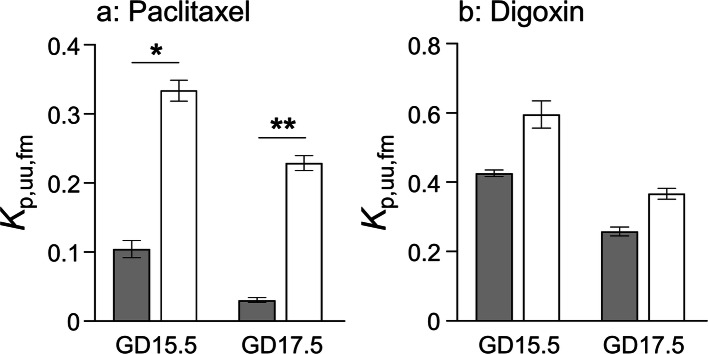
*K*_p,uu,fm_ values of paclitaxel (**A**) and digoxin (**B**) in wild-type (closed bar) and *Mdr1a*^−/−^/*1b*^−/−^ (open bar) mice. Each column represents the mean ± S.E.M. The S.E.M. was calculated according to the law of propagation of error. *p < 0.05, **p < 0.01, significant difference between wild-type and *Mdr1a*^−/−^/*1b*^−/−^ mice in *K*_p,fm_ values.

The *K*_p,uu,fm_ values were determined by multiplying *K*_p,fm_(Table II) and the ratio of *f*_u,fp_ to *f*_u,mp_(Table IV) according to Eq. (2), and are shown in Fig. 3. Since no significant difference in plasma albumin concentration or total protein levels was observed between WT and *Mdr1a/b*^*−/−*^ mice (32), the *K*_p,uu,fm_ value in *Mdr1a/b*^*−/−*^ mice was calculated by using the ratio of *f*_u,fp_ to *f*_u,mp_ determined in WT mice. The *K*_p,uu,fm_ values of paclitaxel and digoxin were all less than unity even in *Mdr1a/b*^*−/−*^ mice, indicating that *CL*_fm,int,all_ is greater than *CL*_mf,int,all_. Thus, another carrier-mediated elimination process from the fetus is assumed to be present for paclitaxel and digoxin.

## Discussion

In this study, we determined the protein amounts of MDR1A and MDR1B at the placental barrier by means of quantitative targeted absolute proteomics and confirmed gestational changes of MDR1A and MDR1B protein expression in the mouse placental labyrinth. The protein amount of MDR1A from GD13.5 to 17.5 remained relatively stable, whereas the MDR1B expression was decreased to almost one-third at GD17.5 (Fig. 2). This result is consistent with previous immunoblot results showing a decline of MDR1 protein expression toward term in both mouse (21–23) and human (33–36). Our results show that the decline in MDR1 level in mice primarily arises from the gestational change in expression of MDR1B, not MDR1A (Fig. 2), and the change appears to be too small to markedly affect drug distribution to the fetus (Fig. 3).

At GD17.5, MDR1A is the dominant MDR1 isoform in the placenta at the protein level, accounting for 80% of total MDR1 (Fig. 2), in accordance with the previous functional observation that the absence of MDR1A in naturally occurring *Mdr1a*-mutants of the CF-1 outbred mouse resulted in enhanced fetal distribution and sensitivity of the fetus to a photoisomer of avermectin B1a (37). On the other hand, the expression of MDR1A is not dominant at earlier gestation days, amounting to 46% and 64% of total MDR1 at GD13.5 and 15.5, respectively (Fig. 2), which is also in good agreement with the previous report of almost equal expression of MDR1A and MDR1B proteins (~0.5 fmol/μg total membrane protein each) in mouse placenta at GD14.5 (8). The fetal distribution of digoxin was reported to be unchanged in pregnant *Mdr1b*^−/−^ mice at GD17.5 (32), but increased fetal drug distribution may be detected in *Mdr1b*^−/−^ mice by using pregnant mice at earlier time points during gestation, with paclitaxel as a more sensitive probe drug compared to digoxin.

The impact of placental MDR1 on fetal distribution, as indicated by the *K*_p,fm_ ratio, differs between paclitaxel and digoxin (Table II). *K*_p,uu,fm_ of digoxin is marginally affected by placental MDR1 while MDR1 decreases *K*_p,uu,fm_ of paclitaxel to less than one-third(Fig. 3). This difference between paclitaxel and digoxin is in accordance with previous observations showing the concentration ratio of fetal tissue to maternal plasma (Supplemental Table S2)(9, 38, 39), but the evaluation using fetal tissue cannot rule out an additional effect of MDR1 expressed in fetal tissues (40). Therefore, the *K*_p,fm_ ratio determined in this study should be a better index for precisely estimating the impact of placental MDR1. The difference between paclitaxel and digoxin is unique to the placental barrier, since the *K*_p,brain_ ratio and *in vitro* MDR1A efflux ratio of digoxin are greater than those of paclitaxel (Table II). The placenta-to-brain and the placenta-to-*in vitro* MDR1A cell ratios of the impact of a single MDR1 molecule, expressed by *R*_P/B_ and *R*_P/C_, respectively, were less than 0.11 for digoxin distribution (Table III). Accordingly, the marginal effect of MDR1 on digoxin fetal distribution cannot be attributed to reduced transport activity of MDR1 protein itself.

Based on the pharmacokinetic model of transport across the mouse placenta (Fig. 1) and the blood-brain barrier (11), *R*_P/B_ can be expressed according to Eq. (10). Similarly, *R*_P/C_ can be expressed according to Eq. (S13) shown in the Supplemental Text. These equations indicate that *R*_P/B_ and *R*_P/C_ are inversely correlated with permeabilities such as *P*_AP2.eff_, *P*_GJ_, and *P*_AP2.inf_. Therefore, the small *R*_P/B_ and *R*_P/C_ values of digoxin can be attributed to the presence of carrier-mediated transport at the apical membrane of SynT-II or gap junction-mediated transfer between SynT-I and SynT-II. It is reasonable to consider that there is a significant contribution of *P*_GJ_ to digoxin transfer, since connexin26 gap junctions are frequently observed along the plasma membrane between SynT-I and SynT-II in transmission electron micrographs of placental labyrinth (5, 6). On the other hand, it seems unlikely that the apical membrane of SynT-II possesses a digoxin transporter that increases *P*_AP2.eff_and/or*P*_AP2.inf_ sufficiently to decrease *R*_P/B_ and *R*_P/C_ to less than 0.11 for following reasons. It is difficult to find a candidate non-MDR1 efflux transporter of digoxin that would increase *P*_AP2.eff_, considering that digoxin is frequently used as a probe for MDR1 in drug interaction studies, and most studies of non-MDR1 digoxin transport have focused on the role of organic anion transporting polypeptides (OATPs) rather than efflux transporters (41). An increase in *P*_AP2. inf_ due to influx transporters such as OATPs would also increase *K*_p,uu,fm_, but in fact the *K*_p,uu,fm_ of digoxin is less than unity in *Mdr1a/b*^*−/−*^ mice (Fig. 3). When *P*_AP2.eff_, *P*_AP2.inf_, *P*_BM1.inf_, and *P*_l.eff_ in Eq. (10) and *P*_AP2.eff_, *P*_AP2.inf_, *P*_BM1.inf_, and *P*_a.eff_ in Eq. (S13)(See Supplemental Text) are assumed to be equal to the permeability due to passive diffusion (*P*_diff_), *R*_P/B_ and *R*_P/C_ can be simply expressed as follows:

14 $$ {R}_{\mathrm{P}/\mathrm{B}}\ \mathrm{and}\ {R}_{\mathrm{P}/\mathrm{C}}=\frac{1}{1+2\frac{P_{\mathrm{GJ}}}{P_{\mathrm{diff}}}} $$

On the basis of this equation, the impact of placental MDR1 on the fetal distribution of drugs would be affected by the permeability through gap junctions between SynT-I and SynT-II and passive diffusion of the drug, and would be greatly decreased when the permeability through the gap junctions exceeds the passive membrane permeability. On the other hand, the permeability through the gap junctions becomes negligible when *R*_P/B_ and *R*_P/C_ are equal to unity.

As regards drugs other than digoxin and paclitaxel, differential impacts of MDR1 on norbuprenorphine distribution into the maternal brain and fetus have been reported in pregnant mice: the fetal tissue-to-maternal plasma AUC ratio was unchanged, but the maternal brain-to maternal plasma AUC ratio was 34-fold higher in *Mdr1a/b*^*−/−*^ mice (8). In addition, impacts of MDR1 on saquinavir distribution into brain and fetus have been independently reported to be similar: the concentration ratio of fetal tissue to maternal plasma was 5- to 7-fold higher in *Mdr1a/b*^*−/−*^ mice at 15 and 30 min after dosing (9) and the brain-to-plasma concentration ratio was 7-fold higher in *Mdr1a*^−/−^ mice at 4 h after dosing (42). Accordingly, it is reasonable to speculate that digoxin and norbuprenorphine have larger permeability through the gap junctions and/or smaller passive diffusion permeability, compared with paclitaxel and saquinavir.

Gap junctions are intercellular channels that allow the transfer of molecules up to 1 kDa, but also larger molecules if they have a linear structure, like peptides and miRNAs (43). The decreased fetal transfer of glucose observed in connexin26-deficient mice (7) supports the idea that connexin26 gap junctions play a key role in the transfer of molecules between SynT-I and SynT-II. According to the crystal structure of the human connexin26 gap junction channel, the amino-terminal helixes of the six subunits line the intracellular pore entrance to form a funnel, whose narrowest region has a diameter of 1.4 nm (44). It is also reported that the connexin26 intercellular channel efficiently transfers small dyes such as Alexa Fluor 350 and Alexa Fluor 488, which have minimal projection diameters calculated using MarvinSketch 21.16.0 (https://chemaxon.com/) of 0.9 nm and 1.3 nm, respectively, but shows a large decrease to 5% in the permeability of a larger dye, Alexa Fluor 594, which has a minimal projection diameter of 1.5 nm (45). The minimal projection diameters of digoxin and norbuprenorphine are calculated to be 1.3 and 1.1 nm, respectively, while those of paclitaxel and saquinavir are both 1.6 nm. Thus, although the molecular weights of these drugs are all less than 1 kDa, the higher permeability of digoxin and norbuprenorphine through connexin26 compared to paclitaxel and saquinavir is consistent with the diameter of these drugs.

Paclitaxel and saquinavir are more lipophilic (log D at pH 7.4 of 6.83 and log P of 4.70, respectively) than digoxin and norbuprenorphine (log D at pH 7.4 of 1.26 and 1.18, respectively) (12, 46). The passive diffusion permeability in parallel artificial membrane permeability assay (PAMPA) at pH 7.4 was reported to be 398 and 114 cm/s for paclitaxel and saquinavir, respectively, which is much larger than the value of 0.8 cm/s for digoxin (47). Taking these data into consideration, digoxin, and presumably norbuprenorphine, would have much larger *P*_GJ_/*P*_diff_ values than paclitaxel and saquinavir, resulting in a lesser impact of placental MDR1 on the fetal distribution of these drugs.

The *K*_p,uu,fm_ values of paclitaxel and digoxin are less than unity even in *Mdr1a/b*^*−/−*^ mice (Fig. 3). Since *K*_p,uu,fm_ is equivalent to the ratio of *CL*_mf,int,all_ to *CL*_fm,int,all_ as shown by Eq. (2), another transporter increasing *CL*_fm,int,all_ could be present in addition to MDR1. According to Eqs. (4) and (6), *PS*_BM2.inf_, *PS*_AP2.eff_, *PS*_BM1.inf_, and *PS*_AP1.eff_ are capable of increasing *CL*_fm,int,all_. *PS*_BM1.inf_ can be eliminated as a candidate for the *CL*_fm,int,all_ increment of digoxin since an increase of *PS*_BM1.inf_ would also increase *R*_P/B_ and *R*_P/C_ according to Eq. (10). It is difficult to identify a candidate non-MDR1 efflux transporter of digoxin, considering that digoxin is commonly used as a probe for MDR1 (41). Therefore, the change of *CL*_fm,int,all_ of digoxin is most likely due to an increase of *PS*_BM2.inf_, though the localization of OATPs at the basal plasma membrane of SynT-II is uncertain. MRP2 accepts paclitaxel as a substrate (48) and exhibits apical localization in epithelial cells of the visceral yolk sac (49). Contributions of these non-MDR1 transporters might also explain why *K*_p,uu,fm_ decreases with advancing gestation (Fig. 3). The fetal digoxin distribution at 1 h after dosing was reportedly increased with advancing gestation (50), but this may simply reflect the gestational increase in exchange surface area per gram of placenta, as observed for other substances (51, 52), which would not affect the steady-state concentration determined in the present study. Further studies are needed to clarify whether OATPs and MRP2 significantly contribute to decrease *K*_p,uu,fm_ of digoxin and paclitaxel, respectively.

The amount of MDR1 protein in the human placental MVM-enriched fraction at term in this study was 0.26 fmol/μg protein (Fig. 2), which is only 4.0% of the estimated MDR1 protein amount at the apical membrane of SynT-II cells in the pregnant mouse at GD17.5 (Table I). The amount of MDR1 observed here is somewhat lower than the reported amount of MDR1 protein (mean: 0.67 fmol/μg protein, mode: ~0.4 fmol/μg protein) in the total membrane fraction from term human placenta (36). However, considering that the majority of the total membrane fraction is MVM membrane (the membrane surface area of MVM is 6-fold greater than that of basal plasma membrane (53)), it is clear that the amount of MDR1 protein in the human placental barrier at term is much lower than that in mice. However, *R*_P/B_ and *R*_P/C_ in human are assumed to be equal to unity, since MDR1 in the human placenta is localized at the MVM of a SynT monolayer (1). For digoxin fetal distribution, the lower protein amount of MDR1 would be reversed, with a high *R*_P/B_ and *R*_P/C_ in human as compared with mice. Thus, assuming a negligible interspecies difference in MDR1-mediated efflux activity of digoxin (25), the human *K*_p,fm_ ratios of digoxin estimated from the *K*_p,brain_ ratio in this study and the *in vitro* MDR1 efflux ratio (11) (Table II) turn out to be almost the same as in mice (~1.4), and are also consistent with the reciprocal of umbilical-to-maternal plasma concentration ratio of digoxin measured at the time of delivery (0.77) (54). On the other hand, the effect of placental MDR1 on the fetal distribution of paclitaxel in humans is expected to be much smaller than that in mice. The human *K*_p,fm_ ratios of paclitaxel estimated from the *K*_p,brain_ ratio in this study and the *in vitro* MDR1 efflux ratio (11) (Table II) are both 1.4.

## Conclusion

The present study demonstrated that murine placental MDR1 has a minimal influence on the fetal distribution of certain substrates, such as digoxin. A pharmacokinetic model including transfer between two SynT layers through connexin26 gap junctions showed that the impact of placental MDR1 is inversely correlated to the ratio of permeability though gap junctions to passive diffusion permeability. Therefore, the fetal transfer of MDR1 substrate drugs with higher gap junction permeability or lower passive diffusion permeability is expected to be only weakly affected by MDR1 due to bypass transfer through gap junctions. This feature is unique to the murine placenta, so care is needed when attempting to predict the human fetal distribution and action of MDR1 substrate drugs with low molecular weight and low lipophilicity from the results of animal experiments using rodents.

## Supplementary Information

ESM 1 (PDF 1.05 MB)

## Abbreviations

*CL*_fm_: Plasmatic clearance in the fetal-to-maternal direction
*CL*_fm,int,all_: Fetus-to-mother intrinsic clearance
*CL*_mf_: Plasmatic clearance in the maternal-to-fetal direction
*CL*_mf,int,all_: Mother-to-fetus intrinsic clearance
*f*_u,fp_: Unbound fraction in the fetal plasma
*f*_u,mp_: Unbound fraction in the maternal plasma
fp: Fetal plasma
GD: Gestational day
IS: Internal standard
*K*_p,brain_: Maternal brain-to-plasmaconcentration ratio
*K*_p,brain_ ratio: Ratio of *K*_p,brain_ in *Mdr1a/b*^*−/−*^to wild type mice
*K*_p,fm_: Fetal-to-maternal plasma concentration ratio
*K*_p,fm_ ratio: Ratio of *K*_p,fp_ in *Mdr1a/b*^*−/−*^to wild type mice
*K*_p,uu,brain_: Unbound plasma concentration ratio of maternal brain to plasma
*K*_p,uu,fm_: Unbound plasma concentration ratio of fetal plasma to maternal plasma
MDR1: Multidrug resistance protein 1
MVM: Microvillous membrane
OATPs: Organic anion transporting polypeptides
*P*_AP2.inf_: Permeability for influx across the apical membrane of SynT-II
*P*_AP2.eff_: Permeability for influx across the apical membrane of SynT-II
*P*_BM1.inf_: Permeability for influx across the basal plasma membrane of SynT-I
*P*_diff_: Permeability due to passive diffusion
*P*_GJ_: Permeability for transfer mediated by gap junctions
*P*_l.eff_: Luminal efflux permeability, excluding MDR1-mediated efflux, at brain capillary endothelial cells
PS: Permeability-surface area
*PS*_AP1.eff_: PS product for efflux across the apical membrane of SynT-I
*PS*_AP1.inf_: PS product for influx across the apical membrane of SynT-I
*PS*_AP2.eff_: PS product for efflux across the apical membrane of SynT-II, excluding MDR1-mediated efflux
*PS*_AP2.inf_: PS product for influx across the apical membrane of SynT-II
*PS*_BM1.eff_: PS product for efflux across the basal plasma membrane of SynT-I
*PS*_BM1.inf_: PS product for influx across the basal plasma membrane of SynT-I
*PS*_BM2.eff_: PS product for efflux across the basal plasma membrane of SynT-II
*PS*_BM1.inf_: PS product for influx across the basal plasma membrane of SynT-II
*PS*_GJ_: PS product for gap junction-mediated transfer between SynT-I and SynT-II
*PS*_l.eff_: PS product for luminal efflux, excluding MDR1-mediated efflux, at brain capillary endothelial cells
*PS*_MDR1,BBB_: PS product for efflux mediated by MDR1 at the blood-brain barrier
*PS*_MDR1,PB_: PS product for efflux mediated by MDR1 at the placental barrier
*R*_P/B_: Placental barrier-to-blood brain barrier conversion ratio of MDR1 contribution per single MDR1 molecule
*R*_P/C_: Placental barrier-to-*in vitro*MDR1A cell conversion ratio of MDR1 contribution per single MDR1 molecule
SRM: Selected reaction monitoring
SynT: Syncytiotrophoblasts
