# Supplementary material for: Limited Impact of Murine Placental MDR1 on Fetal Exposure of Certain Drugs Explained by Bypass Transfer Between Adjacent Syncytiotrophoblast Layers
Source: Pharm Res. 2022 Jan 26;39(7):1645–58. doi: 10.1007/s11095-022-03165-6 (PMC9246986; doi:10.1007/s11095-022-03165-6)
Supplement: Supplementary file 1 — (PDF 1.05 MB) [file 11095_2022_3165_MOESM1_ESM.pdf]

Electronic supplementary materials

Journal: Pharmaceutical Research

Title: Limited Impact of Murine Placental MDR1 on Fetal Exposure of Certain Drugs Explained by Bypass Transfer between Adjacent Syncytiotrophoblast Layers

Authors and affiliations:

Arimi Fujita[1-3], Saki Noguchi[1], Rika Hamada[1], Satoko Inoue[1], Tsutomu Shimada[2,3], Satomi Katakura[4], Tetsuo Maruyama[4], Yoshimichi Sai[2,3] Tomohiro Nishimura[1], Masatoshi Tomi[1]

[1] Division of Pharmaceutics, Faculty of Pharmacy, Keio University, Minato-ku, Tokyo 105-8512, Japan

[2] Department of Clinical Pharmacokinetics, Graduate School of Medical Sciences, Kanazawa University, Kanazawa, Ishikawa 920-8641, Japan

[3] Department of Hospital Pharmacy, University Hospital, Kanazawa University, Kanazawa, Ishikawa 920-8641, Japan

[4] Department of Obstetrics and Gynecology, Keio University School of Medicine, Shinjuku-ku, Tokyo 160-8582, Japan

Corresponding author:

Masatoshi Tomi

tomi-ms@pha.keio.ac.jp

Theoretical development of expressions for CL_{mf} and CL_{fm} in the rodent transplacental pharmacokinetic model for MDR1 substrates (Fig. 1)

Plasmatic clearance in the maternal-to-fetal direction (CL_{mf})

Mass balance rate equations describing the transfer of MDR1 substrates from maternal to fetal plasma and an equation describing CL_{mf} are as follows:

$$\frac{dX_1}{dt} = PS_{\mathrm{AP1.inf}} \times f_{\mathrm{u,mp}} \times C_{\mathrm{mpl}} + PS_{\mathrm{BM1.inf}} \times f_{\mathrm{u,i}} \times C_{\mathrm{i}} + PS_{\mathrm{GJ}} \times f_{\mathrm{u,2}} \times C_2 - \left(PS_{\mathrm{BM1.eff}} + PS_{\mathrm{GJ}} + PS_{\mathrm{AP1.eff}}\right) \times f_{\mathrm{u,1}} \times C_1 \quad (S1)$$

$$\frac{dX_{\mathrm{i}}}{dt} = PS_{\mathrm{BM1.eff}} \times f_{\mathrm{u,1}} \times C_1 + \left(PS_{\mathrm{MDR1,PB}} + PS_{\mathrm{AP2.eff}}\right) \times f_{\mathrm{u,2}} \times C_2 - \left(PS_{\mathrm{BM1.inf}} + PS_{\mathrm{AP2.inf}}\right) \times f_{\mathrm{u,i}} \times C_{\mathrm{i}} \quad (S2)$$

$$\frac{dX_2}{dt} = PS_{\mathrm{AP2.inf}} \times f_{\mathrm{u,i}} \times C_{\mathrm{i}} + PS_{\mathrm{GJ}} \times f_{\mathrm{u,1}} \times C_1 - \left(PS_{\mathrm{BM2.eff}} + PS_{\mathrm{GJ}} + PS_{\mathrm{MDR1,PB}} + PS_{\mathrm{AP2.eff}}\right) \times f_{\mathrm{u,2}} \times C_2 \quad (S3)$$

$$CL_{\mathrm{mf}} = \frac{PS_{\mathrm{BM2.eff}} \times f_{\mathrm{u,2}} \times C_2}{C_{\mathrm{mp}}} \quad (S4)$$

where X_1, C_1, and $f_{\mathrm{u,1}}$ respectively represent the amount, concentration, and unbound fraction of drug in SynT-I, X_{i}, C_{i}, and $f_{\mathrm{u,i}}$ respectively represent those in the intercellular space between SynT-I and SynT-II, and X_2, C_2, and $f_{\mathrm{u,2}}$ respectively represent those in SynT-II. C_{mpl} represents the plasma drug concentration in maternal blood space in the placental labyrinth.

At the steady state, the values given by Eqs. (S1) to (S3) are zero, and C_{mpl} can be replaced with C_{mp} on the assumption that fetal metabolism is negligible. The relationship between $f_{\mathrm{u,1}} \times C_1$ and $f_{\mathrm{u,2}} \times C_2$ at the steady state can be obtained from Eqs. (S2) and (S3) as follows:

$$f_{\mathrm{u,1}} \times C_1 = \frac{PS_{\mathrm{BM2.eff}} + \beta_{21}}{\beta_{12}} \times f_{\mathrm{u,2}} \times C_2 \quad (S5)$$

$$\beta_{12} = PS_{\mathrm{BM1.eff}} \times \frac{PS_{\mathrm{AP2.inf}}}{PS_{\mathrm{BM1.inf}} + PS_{\mathrm{AP2.inf}}} + PS_{\mathrm{GJ}}$$

$$\beta_{21} = \left(PS_{\mathrm{MDR1,PB}} + PS_{\mathrm{AP2.eff}}\right) \times \frac{PS_{\mathrm{BM1.inf}}}{PS_{\mathrm{BM1.inf}} + PS_{\mathrm{AP2.inf}}} + PS_{\mathrm{GJ}}$$

Then the relationship between $f_2 \times C_2$ and $f_{\mathrm{mp}} \times C_{\mathrm{mp}}$ at the steady state can be obtained from Eqs. (S1), (S2), and (S5) as follows:

$$f_{\mathrm{u,2}} \times C_2 = \frac{\beta_{12} \times PS_{\mathrm{AP1.inf}}}{\beta_{12} \times PS_{\mathrm{BM2.eff}} + \beta_{21} \times PS_{\mathrm{AP1.eff}} + PS_{\mathrm{BM2.eff}} \times PS_{\mathrm{AP1.eff}}} \times f_{\mathrm{u,mp}} \times C_{\mathrm{mp}} \quad (S6)$$

Finally, the CL_{mf} at the steady state can be obtained from Eqs. (S4) and (S6) as follows:

$$CL_{\mathrm{mf}} = f_{\mathrm{u,mp}} \times CL_{\mathrm{mf,int,all}}$$
$$CL_{\mathrm{mf,int,all}} = PS_{\mathrm{AP1.inf}} \times \frac{\beta_{12} \times PS_{\mathrm{BM2.eff}}}{\beta_{12} \times PS_{\mathrm{BM2.eff}} + \beta_{21} \times PS_{\mathrm{AP1.eff}} + PS_{\mathrm{BM2.eff}} \times PS_{\mathrm{AP1.eff}}}$$

Plasmatic clearance in the fetal-to-maternal direction (CL_{fm})

Mass balance rate equations describing the transfer of MDR1 substrates from fetal to maternal plasma and an equation describing CL_{fm} are as follows:

$$\frac{dX_2}{dt} = PS_{\mathrm{BM2.inf}} \times f_{\mathrm{u,fp}} \times C_{\mathrm{fpl}} + PS_{\mathrm{AP2.inf}} \times f_{\mathrm{u,i}} \times C_{\mathrm{i}} + PS_{\mathrm{GJ}} \times f_{\mathrm{u,1}} \times C_1 - \left(PS_{\mathrm{BM2.eff}} + PS_{\mathrm{GJ}} + PS_{\mathrm{MDR1,PB}} + PS_{\mathrm{AP2.eff}}\right) \times f_{\mathrm{u,2}} \times C_2 \qquad (S7)$$

$$\frac{dX_{\mathrm{i}}}{dt} = PS_{\mathrm{BM1.eff}} \times f_{\mathrm{u,1}} \times C_1 + \left(PS_{\mathrm{MDR1,PB}} + PS_{\mathrm{AP2.eff}}\right) \times f_{\mathrm{u,2}} \times C_2 - \left(PS_{\mathrm{BM1.inf}} + PS_{\mathrm{AP2.inf}}\right) \times f_{\mathrm{u,i}} \times C_{\mathrm{i}} \qquad (S2)$$

$$\frac{dX_1}{dt} = PS_{\mathrm{BM1.inf}} \times f_{\mathrm{u,1}} \times C_1 + PS_{\mathrm{GJ}} \times f_{\mathrm{u,2}} \times C_2 - \left(PS_{\mathrm{BM1.eff}} + PS_{\mathrm{GJ}} + PS_{\mathrm{AP1.eff}}\right) \times f_{\mathrm{u,1}} \times C_1 \qquad (S8)$$

$$CL_{\mathrm{fm}} = \frac{PS_{\mathrm{AP1.eff}} \times f_{\mathrm{u,1}} \times C_1}{C_{\mathrm{fp}}} \qquad (S9)$$

where C_{fpl} represents the plasma drug concentration in fetal vessels in the placental labyrinth.

At the steady state, the values given by Eqs. (S2), (S7), and (S8) are zero and C_{fpl} can be replaced with C_{fp} on the assumption that fetal metabolism is negligible. So, the relationship between$f_{\mathrm{u,2}} \times C_2$ and $f_{\mathrm{u,1}} \times C_1$ at the steady state can be obtained from Eqs. (S2) and (S8) as follows:

$$f_{\mathrm{u,2}} \times C_2 = \frac{PS_{\mathrm{AP1.eff}} + \beta_{12}}{\beta_{21}} \times f_{\mathrm{u,1}} \times C_1 \qquad (S10)$$

Then the relationship between $f_{\mathrm{u,1}} \times C_1$ and $f_{\mathrm{u,fp}} \times C_{\mathrm{fp}}$ at steady state can be obtained from Eqs. (S2), (S7), and (S10) as follows:

$$f_{\mathrm{u,1}} \times C_1 = \frac{\beta_{21} \times PS_{\mathrm{BM2.inf}}}{\beta_{12} \times PS_{\mathrm{BM2.eff}} + \beta_{21} \times PS_{\mathrm{AP1.eff}} + PS_{BM2.\mathrm{eff}} \times PS_{\mathrm{AP1.eff}}} \times f_{\mathrm{u,fp}} \times C_{\mathrm{fp}} \qquad (S11)$$

Finally, the CL_{fm} can be obtained from Eqs. (S9) and (S11) as follows:

$$CL_{\mathrm{fm}} = f_{\mathrm{u,fp}} \times CL_{\mathrm{fm,int,all}}$$
$$CL_{\mathrm{fm,int,all}} = PS_{\mathrm{BM2.inf}} \times \frac{\beta_{21} \times PS_{\mathrm{AP1.eff}}}{\beta_{12} \times PS_{\mathrm{BM2.eff}} + \beta_{21} \times PS_{\mathrm{AP1.eff}} + PS_{\mathrm{BM2.eff}} \times PS_{\mathrm{AP1.eff}}}$$

Evaluation of the contribution of MDR1 to drug distribution at the placental barrier from data in an MDR1-expressing cell monolayer

The *in vitro* MDR1 efflux ratio determined using cells apically expressing MDR1 and mock cells is given by

$$\textit{In vitro}\ \text{MDR1 efflux ratio} = 1 + \frac{PS_{\mathrm{MDR1},\textit{in vitro}}}{PS_{\mathrm{a.eff}}} \qquad (S12)$$

where $PS_{\mathrm{MDR1},\textit{in vitro}}$ and $PS_{\mathrm{a.eff}}$ represent the PS product for the efflux mediated by MDR1 and the apical efflux excluding MDR1-mediated efflux, respectively, in the monolayer of MDR1-expressing cells. Accordingly, the contribution of MDR1 to fetal drug distribution, expressed as $K_{\mathrm{p,fm}}\ \text{ratio} - 1$, per single MDR1 protein at the placental barrier can be obtained from $\textit{In vitro}\ \text{MDR1 efflux ratio} - 1$ per single MDR1 protein using the conversion ratio ($R_{\mathrm{P/C}}$) given by

$$R_{\mathrm{P/C}} = \frac{\dfrac{PS_{\mathrm{MDR1,PB}}}{PS_{\mathrm{AP2.eff}} + PS_{\mathrm{GJ}} \times \left(1 + \dfrac{PS_{\mathrm{AP2.inf}}}{PS_{\mathrm{BM1.inf}}}\right)} \times \dfrac{1}{\substack{\text{MDR1 protein amounts} \\ \text{in apical side of SynT-II (fmol/μg protein)}}}}{\dfrac{PS_{\mathrm{MDR1},\textit{in vitro}}}{PS_{\mathrm{a.eff}}} \times \dfrac{1}{\substack{\text{MDR1 protein amounts} \\ \text{in apical side of MDR1 expressing cell monolayer (fmol/μg protein)}}}}$$
$$= \frac{P_{\mathrm{a.eff}}}{P_{\mathrm{AP2.eff}} + P_{\mathrm{GJ}} \times \left(1 + \dfrac{P_{\mathrm{AP2.inf}}}{P_{\mathrm{BM1.inf}}}\right)} \qquad (S13)$$

where $P_{\mathrm{a.eff}}$ represents the apical efflux permeability excluding MDR1-mediated efflux.

When $P_{\mathrm{AP2.eff}}$, $P_{\mathrm{AP2.inf}}$, $P_{\mathrm{BM1.inf}}$, and $P_{\mathrm{a.eff}}$ in Eq. (S13) are assumed to be equal to P_{diff}, $R_{\mathrm{P/C}}$ can be simply expressed as follows:

$$R_{\mathrm{P/C}} = \frac{1}{1 + 2\frac{P_{\mathrm{GJ}}}{P_{\mathrm{diff}}}} \tag{14}$$

Estimated expression amounts of MDR1 in the apical membrane of SynT-II at mouse placenta and the luminal membrane at mouse brain capillary endothelial cells

The total amount of MDR1 in the mouse placental labyrinth was calculated to be lower at GD17.5 (0.82 fmol/μg protein) than at GD13.5 (0.93 fmol/μg protein) or 15.5 (1.25 fmol/μg protein), primarily due to the decline of MDR1B protein expression. The amount of MDR1 at the apical membrane of SynT-II can be roughly estimated to be 8 times that in the labyrinth based on the assumption that approximately one-eighth of the plasma membrane fraction prepared from the placental labyrinth is derived from the apical membrane of SynT-II cells expressing MDR1. This assumption is plausible because the labyrinth is comprised of four cell layers, i.e., the continuous but fenestrated trophoblast giant cell layer lining the maternal sinusoids, two syncytial trophoblast layers (SynT-I and SynT-II), and the endothelial cell layer of the fetal capillaries, and SynT-II is subdivided into the slightly undulating but not microvillous apical membrane and the basal membrane (6).

The expression of MDR1 in the luminal membrane of brain capillaries is estimated to be twice that in the plasma membrane fraction of brain capillaries, that consists of the luminal and the abluminal membranes, reported by Uchida et al. (2011) (11).

Supplemental Table S1

Protein expression levels of MDR1/MDR1A and MDR1B in individual donors (human placental MVMs) or samples (mouse plasma membrane enriched fractions)

Protein	Q1 > Q3		Protein expression levels (fmol/µg protein) in mouse plasma membrane fraction									Protein expression levels (fmol/µg protein) in human placental MVM-enriched fraction			
	ST peptide (m/z)	IS peptide (m/z)	GD13.5			GD15.5			GD17.5						
			#1	#2	#3	#1	#2	#3	#1	#2	#3	#1	#2	#3	#4
MDR1/ MDR1A	467.8 > 719.4	471.3 / 726.4	0.149	0.487	0.549	0.194	1.166	1.297	1.254	0.467	0.482	0.330	0.293	0.198	0.311
	467.8 > 216.1	471.3 / 216.1	0.176	0.384	0.511	0.161	0.952	1.071	0.790	0.424	0.426	0.352	0.293	0.231	0.253
	467.8 > 561.3	471.3 / 568.3	0.174	0.529	0.904	0.263	0.991	1.124	1.014	0.485	0.534	0.263	0.172	0.157	0.246
	**Mean**		**0.166**	**0.467**	**0.655**	**0.206**	**1.036**	**1.164**	**1.019**	**0.459**	**0.481**	**0.315**	**0.253**	**0.196**	**0.270**
	**S.E.M.**		**0.009**	**0.043**	**0.130**	**0.030**	**0.066**	**0.068**	**0.130**	**0.018**	**0.031**	**0.027**	**0.040**	**0.021**	**0.021**
MDR1B	524.8 > 848.5	528.8 / 856.5	0.155	0.416	1.038	0.148	0.443	0.586	0.117	0.306	0.116				
	524.8 > 735.4	528.8 / 743.4	0.220	0.468	1.144	0.227	0.385	0.619	0.144	0.211	0.207				
	524.8 > 517.3	528.8 / 525.3	0.117	0.412	0.566	0.351	0.604	0.689	0.131	0.023	0.204				
	**Mean**		**0.164**	**0.432**	**0.916**	**0.242**	**0.478**	**0.631**	**0.131**	**0.180**	**0.176**				
	**S.E.M.**		**0.030**	**0.018**	**0.180**	**0.059**	**0.066**	**0.031**	**0.008**	**0.083**	**0.030**				

Supplemental Table S2

Concentration ratio of fetal tissue-to-maternal plasma and brain-to-plasma in wild-type (WT) and *Mdr1a*$^{-/-}$/*1b*$^{-/-}$ or *Mdr1a*$^{-/-}$ (KO) mice

Drug	Tissue	Fold increase in KO compared to WT	Time after administration (h)	Reference
Paclitaxel	Fetus	16	1	(9)
	Brain	5.5	8	(39)
Digoxin	Fetus	2.2 and 2.4	4 and 24	(9)
	Brain	19	4	(38)

Pregnant *Mdr1a*$^{-/-}$/*1b*$^{-/-}$ mice at GD15 and non-pregnant *Mdr1a*$^{-/-}$ mice were used for determining concentration ratio of fetal tissue-to-maternal plasma and brain-to-plasma, respectively.

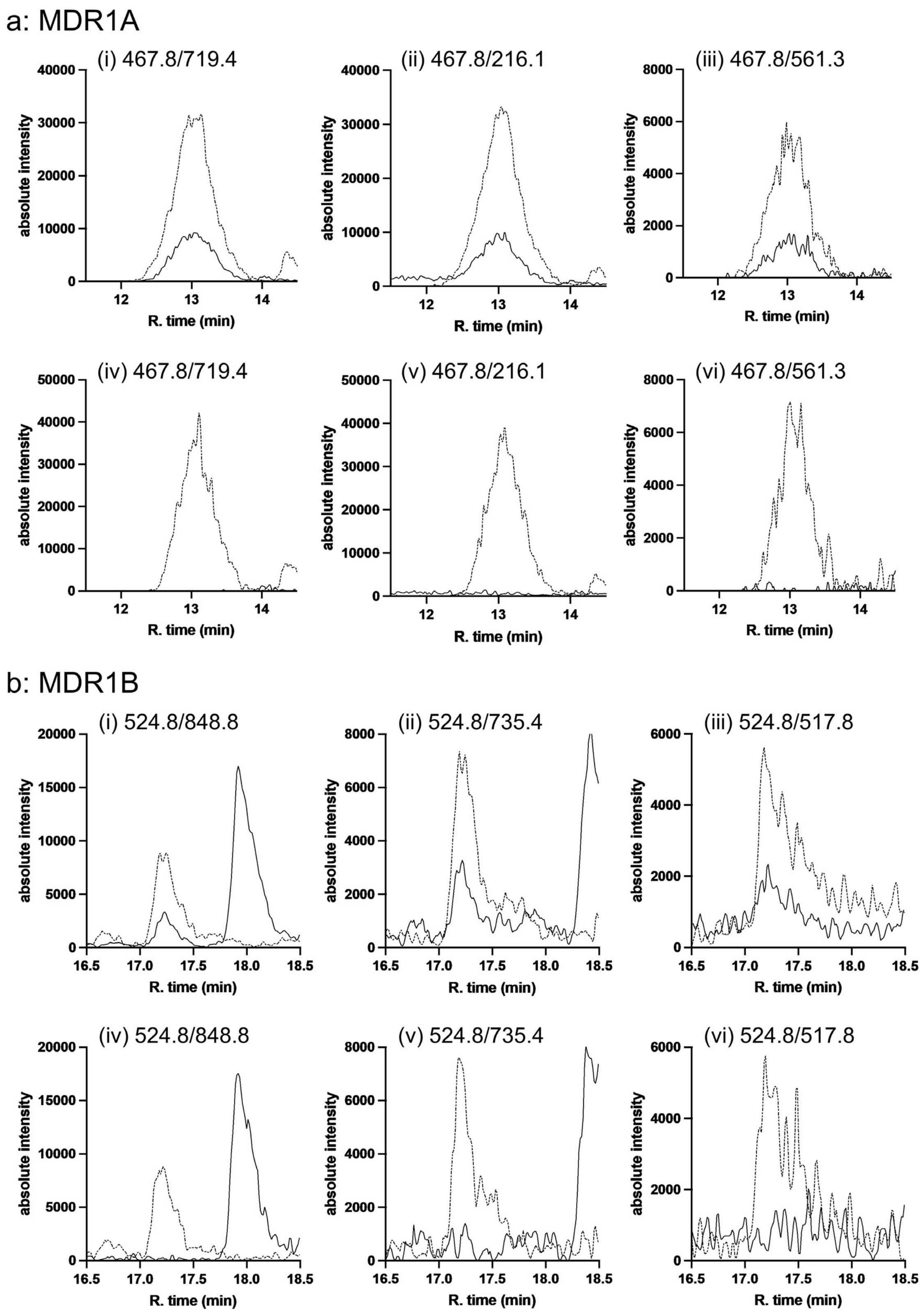

Fig. S1 Peak chromatogram of three SRM/MRM transitions for MDR1A (a) and MDR1B (b) specific peptides, NTTGALTTR and TVIAFGGQQK, respectively. Tryptic digests of plasma membrane fraction from wild-type (i-iii) and *Mdr1a*$^{-/-}$/*Mdr1b*$^{-/-}$ mice (iv-vi) placental labyrinth spiked with internal standard (IS) peptides were subjected to LC-MS/MS. Chromatograms of each sample and the corresponding IS peptide are represented by solid and dashed lines, respectively. Data are the mean from three independent placental preparations.

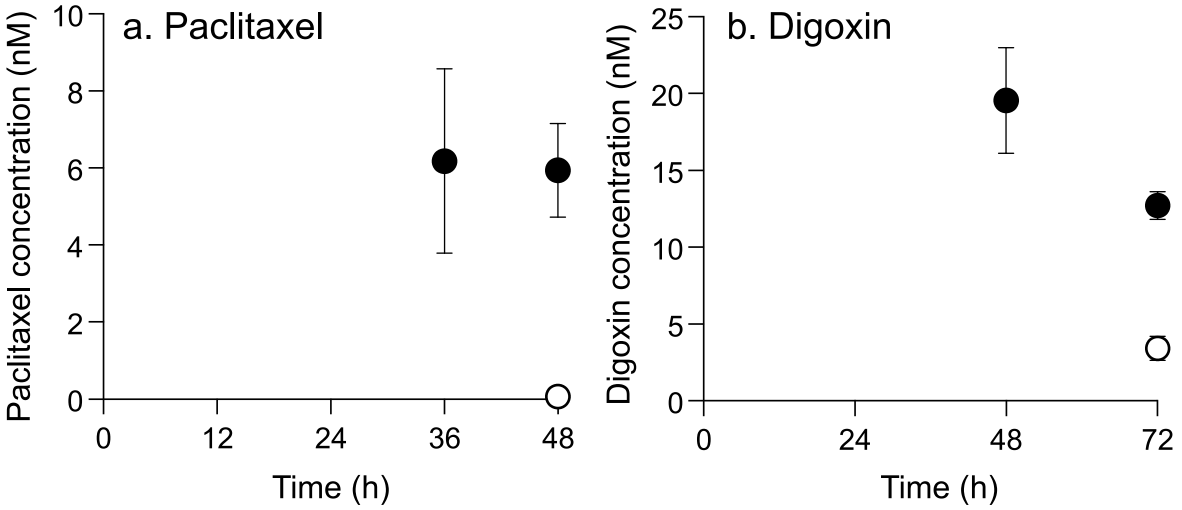

Fig. S2 Plasma concentration-time profiles of paclitaxel (a) and digoxin (b) in wild-type mice during continuous infusion via an osmotic pump for 48 h (paclitaxel) or 72 h (digoxin) to GD17.5. Maternal and fetal plasma concentrations are represented by closed and open circles, respectively. Each point represent the mean ± S.E.M. (n = 3-9).
